# Multivariate hierarchical Bayesian model for differential gene expression analysis in microarray experiments

**DOI:** 10.1186/1471-2105-9-S1-S9

**Published:** 2008-02-13

**Authors:** Hongya Zhao, Kwok-Leung Chan, Lee-Ming Cheng, Hong Yan

**Affiliations:** 1 Department of Electronic Engineering, City University of Hong Kong, Kowloon, Hong Kong; 2 School of Electrical and Information Engineering, University of Sydney, NSW 2006, Australia

## Abstract

**Background:**

Identification of differentially expressed genes is a typical objective when analyzing gene expression data. Recently, Bayesian hierarchical models have become increasingly popular to solve this type of problems. These models show good performance in accommodating noise, variability and low replication of microarray data. However, the correlation between different fluorescent signals measured from a gene spot is ignored, which can diversely affect the data analysis step. In fact, the intensities of the two signals are significantly correlated across samples. The larger the log-transformed intensities are, the smaller the correlation is.

**Results:**

Motivated by the complicated error relations in microarray data, we propose a multivariate hierarchical Bayesian framework for data analysis in the replicated microarray experiments. Gene expression data are modelled by a multivariate normal distribution, parameterized by the corresponding mean vectors and covariance matrixes with a conjugate prior distribution. Within the Bayesian framework, a generalized likelihood ratio test (GLRT) is also developed to infer the gene expression patterns. Simulation studies show that the proposed approach presents better operating characteristics and lower false discovery rate (FDR) than existing methods, especially when the correlation coefficient is large. The approach is illustrated with two examples of microarray analysis. The proposed method successfully detects significant genes closely related to the experimental states, which are verified by the biological information.

**Conclusions:**

The multivariate Bayesian model, compatible with the dependence between mean and variance in the univariate Bayesian model, relaxes the constant coefficient of variation assumption between measurements by adding a covariance structure. This model improves the identification of differentially expressed genes significantly since the Bayesian model fit well with the microarray data.

## Background

DNA microarrays offer a powerful and effective technology to monitor the alterations of gene expression for thousands of genes simultaneously. This technology has been widely applied to the exploration of quantitative changes in gene expression in a variety of areas including diseases and toxicological studies [1-4]. One of the key tasks of microarray analysis is to investigate the expression patterns from the different experiment designs so that differentially expressed (DE) genes can be identified [5,6].

In this paper, we consider the analysis of a two-color cDNA microarray experiment. Briefly, mRNA contained in each of two cell populations is extracted, reverse-transcribed into cDNA, and labelled with either Cy3 (green) or Cy5 (red) dyes. Cy3 and Cy5 preparations are combined and deposited on the microarray, where labelled molecules hybridize to the spots containing their complementary sequence. The amount of hybridization to each spot is quantified by scanning the array with a laser beam and observed the intensities of light emitted [7]. A pair of measurements, separately for the two dyes, are observed as *x*_*gi *_and *y*_*gi *_(*g *= 1,⋯,*N*; *i *= 1,⋯,*n*) for gene *g *on array *i*, where *N *is the number of genes represented on the microarray and *n *is the number of replicated arrays.

Given the microarray expression data, a common task is to determine which genes are differentially expressed under the two conditions. There has been a considerable amount of work in this area [8-26]. The simplest way to ascertain a gene's differential expression is based on a fold change criteria, defined by the log-ratio (log_2_(*x*_*gi*_/*y*_*gi*_)). The straightforward fold-change method widely used by biologists takes into account only the genes whose fold changes are more than 2-fold as differentially expressed genes. The 2-fold rule is too simple to deal with the issues raised by the complicated error in DNA microarray data analysis [8-12].

Traditional statistical methods may not produce reliable results when they are used directly to determine differentially expressed genes. Firstly, it is common to have thousands of genes on one chip with relatively few replications in microarray experiments. Thus, the variance estimates of gene expression data are often unreliable with the small sample size. The common approach using t- or F-statistic is not applicable since it strongly depends on the sample size and normality of the expression data [8-10]. It is known that microarray data may not follow a normal distribution or even be symmetrical and the sample size is generally small [12-16]. Modified t-statistic is suggested by adding a small constant to the gene-specific variance estimate [17]. The method makes the gene-specific variance estimates shrink towards a common variance. Recently, the hierarchical Bayesian models are employed to variance regulation by estimating moderate variances of individual genes [18-26]. The adjusted variances are calculated with the weighted averages of the gene-specific sample variances and pooled variances across all genes. With the additional combination of variances, the performance of these methods is improved significantly in identifying the significance of gene expression.

**Figure 1:**
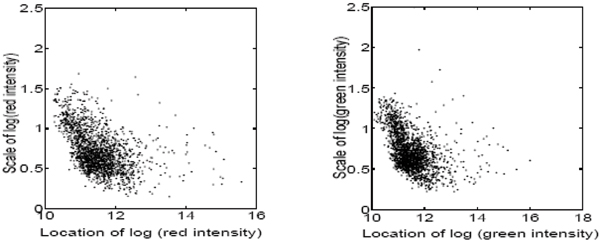
**Robust locations v.s. scales of gene expression data**. The x-axis is the estimation of locations and y-axis is the estimation of scales. The left graph is plotted with the expression data of control groups and the right one is for the Cd toxic treatment (right) groups in Cd toxic microarray experiment.

**Figure 2:**
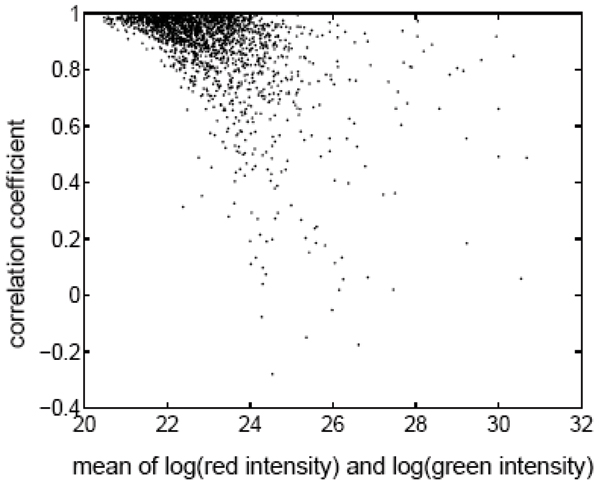
**Mean of log-transformed intensities v.s. correlation coefficient**. The x-axis is the mean of log-transformed measurements over the replications and y-axis is the correlation coefficient between the pair of measurements within a spot.

Another common feature of microarray data is the distinctive error structure with gene variances changing with the expression levels in a nonlinear fashion [14,15]. Their relations are shown with our experimental data in Figure 1. Some traditional methods are statistically inefficient because of the significant violation from the general assumptions. However, the Bayesian philosophy appears to be suitable for this type of problems [18-26]. Instead of the directly modelling the fluctuation of microarray data, Bayesian models are characterized by mixing measurements over a latent gene-specific component. A hierarchical gamma-gamma (GG) model is developed in [18] for detecting changes of gene expression in a two-channel cDNA microarray experiment. The model is extended to replicated chips with multiple conditions using a hierarchical lognormal-normal (LNN) model [26]. Both of them are based on the assumption of a constant coefficient of variation (CV) across genes. According to extensive exploratory data analysis, however, we observe that there are specific correlations between the pair of measurements within each gene spot across samples. The correlation pattern is presented in Figure 2.

Motivated by these error relations, we propose a novel multivariate Bayesian framework for microarray analysis. The multivariate Bayesian model, compatible with the dependence between mean and variance in the univariate Bayesian model, can relax the constant assumption between measurements by adding a covariance structure. Due to the computational complexity within the Bayesian framework, we apply the modified generalized likelihood ratio test (GLRT) proposed by Benjamini and Hochberg [27,28] to detect gene expression patterns. When the Bayesian model is in accordance with the microarray data, the power of true identification of differentially expressed genes can be improved substantially.

In this paper, we describe the multivariate Bayesian hierarchical model for gene expression data analysis, and present the generalized likelihood ratio test (GLRT) procedures with the p-value adjustment to identify differentially expressed genes. The sample size of microarray data play an important role in replicated microarray experiments. So in our simulation study, we first explore the effect of the number of replications in our methodology and suggest that the number of replicated chips is not less than 4. We also compare our methods with existing ones, such as fold change, modified t-test and LNN model. The new methodology shows good performance based on operating characteristics. In the analysis of the real microarray data, our method is proven to be powerful to identify more significant genes.

## Results

### Multivariate hierarchal Bayesian model and inference

Based on the LNN hierarchical model [26], we develop a multivariate model to relax the constant CV assumption between measurements by adding covariance. The model is also compatible with the complicated structure of variance in microarray data. The model is first described in this section, and then the GLRT is employed to infer the expression pattern.

First, we consider the typical two-color microarray data *x*_*gi *_and *y*_*gi *_(*g *= 1,⋯,*N*; *i *= 1,⋯,*n*) for gene *g *on array *i*, where *N *is the number of genes represented on the microarray and *n *is the number of replicated arrays. We denote the *n *replicated pairs of expression levels of gene *g *as **Z**_*g *_= (**z**_*g*1_,⋯,**z**_*gn*_)', where **z**_*gi *_= (*x*_*gi*_, *y*_*gi*_)'. Firstly, *z*_*gi *_is assumed to follow approximately a multivariate Gaussian distribution *N*_2_(**z**_*gi *_| **μ**_*g*_, Σ_*g*_) with a latent gene-specific expression component *π*(**μ**_*g*_, Σ_*g*_). Thus, the likelihood of gene *g *is written as

$$f(Z_{g})=\prod_{i=1}^{n}p(z_{gi}|\mu_{g},\sum_{g})={\left(2\pi \right)}^{-n}{\left|\sum_{g}\right|}^{-\frac{n}{2}}\exp\left\{-\frac{1}{2}\sum_{i}\left(z_{gi}-\mu_{g})'\sum_{g}^{-1}(z_{gi}-\mu_{g}\right)\right\}$$

The Bayesian formulation requires a prior distribution *π*(**μ**_*g*_, Σ_*g*_). For a normal distribution, several kinds of priors for the mean and variance variables have been studied in the literature, including the vague prior and natural conjugate prior [29]. For microarray data, the conjugate prior is a suitable choice [18-22]. Indeed, not only their posterior has the same functional form as the prior, but the conjugate prior also incorporates the inherent dependence between the mean and the variance [26]. The multivariate conjugate prior distribution *π*(**μ**_*g*_, Σ_*g*_) of Equation (1) is composed of the probability distributions of **μ**_*g *_| Σ_*g *_following a multivariate normality and Σ_*g *_following an inverse Wishart distribution (IW) as

$$\begin{array}{cc} \mu_{g}|\sum_{g}\sim N_{2}(\mu_{g};\mu_{g}^{0},\sum_{g}/\lambda_{0}) & \sum_{g}\sim IW(\sum_{g};\nu_{0},\Lambda_{0}^{-1}) \end{array}$$

in which **α **= {*λ*_0_, *ν*_0_, Λ_0_} contains the global hyperparameters and $\Theta =\{\mu_{g}^{0}:g=1,\cdots ,N\}$ the gene-specific parameters. Given the parameters, the conjugate prior $\pi (\mu_{g},\sum_{g})=\pi (\mu_{g},\sum_{g}|\alpha ,\mu_{g}^{0})$ is the following product

$$\pi (\mu_{g},\sum_{g}|\alpha ,\mu_{g}^{0})=N_{2}(\mu_{g};\mu_{g}^{0},\sum_{g}/\lambda_{0})IW(\sum_{g};\nu_{0},\Lambda_{0}^{-1})$$

By the Bayes rule, the corresponding posterior distribution also has the same functional form as the prior

$$\pi (\mu_{g},\sum_{g}|Z_{g},\alpha ,\mu_{g}^{0})=N_{2}(\mu_{g};\mu_{g}^{n},\sum_{g}/\lambda_{n})IW(\sum_{g};\nu_{n},\Lambda_{n}^{-1})$$

where

$$\begin{array}{c} \mu_{g}^{n}=\frac{\lambda_{0}}{\lambda_{0}+n}\mu_{g}^{0}+\frac{n}{\lambda_{0}+n}m_{g}\\ \lambda_{n}=\lambda_{0}+n\\ \nu_{n}=\nu_{0}+n\\ \Lambda_{n}=\Lambda_{0}+\sum_{i=1}^{n}\left(z_{gi}-m_{g})(z_{gi}-m_{g}\right)'+\frac{\lambda_{0}n}{\lambda_{0}+n}(m_{g}-\mu_{g}^{0})(m_{g}-\mu_{g}^{0})' \end{array}$$

and $m_{g}=\sum_{i=1}^{n}z_{gi}/n=(m_{g1},m_{g2})'$, the estimation of mean expression of gene *g *over *n *replications. Obviously, the posterior combines the information from the prior and the data in a sensible way.

Since **α **and Θ are generally unknown, we estimate them with maximum likelihood estimation (MLE). The likelihood functions of gene *g *and over all genes are respectively written as

$$L_{g}(\alpha ,\mu_{g}^{0})=\prod_{g=1}^{N}\phi (z_{gi}|\alpha ,\mu_{g}^{0})=\Phi (z_{g}|\alpha ,\mu_{g}^{0})$$

and

$$L(\alpha ,\Theta )=\prod_{g=1}^{N}L_{g}(\alpha ,\mu_{g}^{0})=\prod_{g=1}^{N}\Phi (z_{g}|\alpha ,\mu_{g}^{0})$$

The gene-specific parameters $\mu_{g}^{0}$ are only related to *L*_*g*_(**α**, $\mu_{g}^{0}$). Their optimal values might be obtained by solving the equation

$$\partial \Phi (Z_{g}|\alpha ,\mu_{g}^{0})/\partial \mu_{g}^{0}=0$$

Therefore, Equation (4) can be rewritten by the Bayes rule as

$$\pi (\mu_{g},\Sigma_{g}|Z_{g},\alpha ,\mu_{g}^{0})$$

With Equations (1), (2), and (3), Equation (6) can be explicitly expressed as

$$\Phi (Z_{g}|\alpha ,\mu_{g}^{0})={\left(2\pi \right)}^{-n}\frac{\sqrt{\lambda_{0}}{\left|\Lambda_{0}\right|}^{\nu_{0}/2}\Gamma \left(\frac{\nu_{n}}{2}\right)\Gamma \left(\frac{\nu_{n}-1}{2}\right)}{\sqrt{\lambda_{n}}{\left|\Lambda_{n}\right|}^{\nu_{n}/2}\Gamma \left(\frac{\nu_{0}}{2}\right)\Gamma \left(\frac{\nu_{0}-1}{2}\right)}$$

Finally, the following solutions are calculated as the estimates of $\mu_{g}^{0}$

$$\begin{array}{cc} {\hat{\mu }}_{g}^{0}=m_{g}=(m_{g1},m_{g2})', & g=1,\cdots ,N \end{array}$$

Given these estimates ${\hat{\mu }}_{g}^{0}$, the global parameters $\hat{\alpha }$ can be estimated by maximizing the likelihood function in Equation (5).

Based on the proposed multivariate hierarchical model, the GLRT, which is a generalization of the Neyman-Pearson test, can be used for the identification. In fact, the identification between two cell populations is equivalent to testing the following hypothesis,

$$\begin{array}{ccc} H_{0}:\mu_{g1}^{0}=\mu_{g2}^{0} & v.s. & H_{1}:\mu_{g1}^{0}\neq \mu_{g2}^{0} \end{array}$$

Thus, the corresponding GLRT statistic for our hypothesis can be defined as follows:

$$\kappa_{g}=2\ln\left(\frac{\max_{\mu_{g1}^{0},\mu_{g2}^{0}}L_{g}(\hat{\alpha },{\hat{\mu }}_{g}^{0})}{\max_{\mu_{g1}^{0}=\mu_{g2}^{0}}L_{g}(\hat{\alpha },{\hat{\mu }}_{g}^{0})}\right)$$

Obviously, the denominator of Equation (8) is the maximization subject to $\mu_{g1}^{0}=\mu_{g2}^{0}=u_{g}^{0}$ while the optimization in the numerator is unconstrained. In fact, the theoretical optimal estimates of $\mu_{g}^{0}$ without constraint are determined in Equation (7). Also the estimates with the constraint can be found by solving

$$\{\begin{array}{l} \partial \Phi (Z_{g}|\alpha ,\mu_{g}^{0})/\partial \mu_{g}^{0}=0\\ \begin{array}{cc} s.t. & \mu_{g1}^{0}= \end{array}\mu_{g2}^{0} \end{array}$$

and the solutions are

$$\begin{array}{cc} {\hat{u}}_{g}^{0}=\frac{m_{g1}(\Lambda_{22}^{0}-\Lambda_{21}^{0}+\Delta_{22}^{g}-\Delta_{21}^{g})+m_{g2}(\Lambda_{11}^{0}-\Lambda_{12}^{0}+\Delta_{11}^{g}-\Delta_{12}^{g})}{\left(\Lambda_{22}^{0}-\Lambda_{21}^{0}+\Delta_{22}^{g}-\Delta_{21}^{g})+(\Lambda_{11}^{0}-\Lambda_{12}^{0}+\Delta_{11}^{g}-\Delta_{12}^{g}\right)} & g=1,\cdots ,N \end{array}$$

It is proven that *κ*_*g *_approximately follows the *χ*^2 ^distribution with one degree of freedom on the null hypothesis [28]. If *κ*_*g *_is larger than some critical value *κ *of *χ*^2^(1), we would not reject the alternative H_1_, that is to say, gene *g *would be identified as DE gene, otherwise as an equivalently expressed (EE) gene. However, it is essential to control some erroneous rejections and acceptances in testing situation. In the context of microarray, the false discovery rate (FDR) has emerged as a practical object to be controlled in multiple testing [30,31]. The FDR is defined as the expectation of type I errors among the rejected null hypothesis, that is, the average of the ratios of the number of false positives to the number of DE genes identified. The scheme of Benjamini and Hochberg (BH-method) is applied to adjust p-value in the testing of microarray data [21,27] (see section "multiple testing").

### Simulation studies

The purpose of our simulation study is to determine the effect of sample size in our model and compare the proposed method with classical statistics for microarray data analysis. We simulate the expression data with *N *= 2000 genes and *n *= 6 replications generated using our model. Different expression patterns are simulated by adjusting the element values of $\mu_{g}^{0}$. For example, EE genes are generated with the same value $\mu_{g1}^{0}=\mu_{g2}^{0}$; DE genes are obtained with different values uniformly sampled from different intervals to make $\mu_{g1}^{0}\neq \mu_{g2}^{0}$. The probability of differential expression is set to *p *= 0.05 for the binomial distribution to select the DE genes.

Microarray data are typically "large *N *and small *n*", that is, the number of samples is much smaller than the number of genes. Especially with the emergence of replicated microarray, the number of replication is always discussed in microarray analysis [9-11]. Multiple testing is always employed in microarray analysis [30,31]. However, multiple testing is generally distorted by the dimension curse, which makes parameter estimates biased with a smaller number of sample sizes. On the other hand, a larger number of genes appear to compensate partially for the destabilizing effect of the sample size, especially for the estimation of the common parameters of all genes. So we should explore the effect of the sample size in our methods. We simulate the replicated measurements with the previous steps, only changing the number of replication from *n *= 2 to 12. Then we estimate the corresponding parameters of our hierarchical model and calculate the statistic *κ*_*g *_(*g *= 1,⋯,2000) respectively for the 11 data sets.

**Figure 3:**
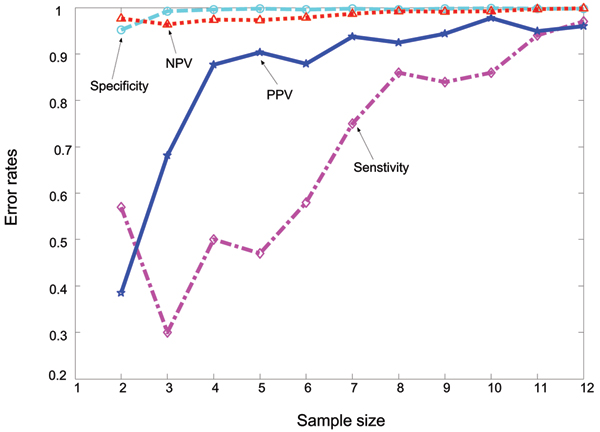
**Effect of sample size on error rates in the multiple testing in our simulation study**. Effect of the sample size on error in multiple testing with the GLRT to identify the DE genes under the multivariate hierarchical model. The x-axis is the number of replications and the y-axis is the error rates. The sensitivity is expressed by diamond, specificity by circle, PPV by star and NPV by triangle.

Knowing the underlying expression of each gene, we compute several corresponding statistics of error rates, including as sensitivity, specificity, positive predict value (PPV) and negative predict value (NPV), which are defined in the subsection "Multiple testing" of the section "Methods", for data sets of different sample sizes. The results are plotted in Figure 3, where the x-axis represents the number of replications and the y-axis represents different error rates. Obviously, the sample size shows little effect on NPV and specificity but significant effect on PPV and sensitivity. All of them almost increase and approximate to stability when the sample sizes increase, so the performance of the method becomes better and better. It is discovered that the PPV of our testing is significantly increased when the sample size *n *= 4. So in part it is reasonable to select *n *= 4, as suggested in literature [9].

In the simulation studies, we also compare our methodology with existing methods for microarray data analysis, such as the fold-change, t-test and LNN model. The fold-change method makes use of direct comparison of intensities, in which the error structure is ignored. The two-sample t-test overcomes the limitation by assuming the normality of expression data, but it is affected by the normality assumption and sample size. The LNN Bayesian model is developed to address these shortcomings. We improve the LNN model using the multivariate Bayesian model by considering the correlation between two measurements of each spot. GLRT is applied to test the hypotheses within the multivariate Bayesian framework.

Flexibility in modelling the correlation between the measurements is a key advantage of the proposed method. Thus, the expression data can be simulated with different correlation coefficients. In fact, Figure 3 shows the simulated data for *ρ *= 0.9. In order to evaluate the performance, we also simulate two data sets with 0.1 and 0.5. Knowing the true expression patterns of each gene, we can calculate the error rates of inference such as sensitivity, specificity, PPV, NPV and FDR that are defined in the section "multiple testing". The results are also shown in Table 1. In comparison with the good performance of GLRT, t-test is poor in sensitivity, PPV and FDR. With the increase of the correlation coefficient *ρ*, the rates of true identification of DE in t-test fall from 0.56 to 0.41 and from 0.67 to 0.57 in the LNN model. On the contrary, the rate in our model is increased from 0.74 to 0.98. That implies that the LNN model and the t-test do not perform well for highly dependent observations. As to the fold-change method, both sensitivity and FDR are very low and their ranges are (0.14, 0.31) and (0, 0.03) respectively. In fact, the number of DE genes identified with fold-change is reasonably small in comparison with the large number of genes being tested. The results show that the fold-change method can be too conservative, that the performance of t-test can be misleading, and that the identification capability of the LNN model is limited especially when the assumption is deviated.

**Table 1:** Operating characteristics in simulation study of *ρ *= 0.1, 0.5 and 0.9.

Corr. Coef.	Method	Sensitivity	Specificity	PPV	NPV	FDR
0.1	GLRT	0.742	0.998	0.960	0.987	0.040
	LNN	0.667	0.991	0.826	0.980	0.174
	t-test	0.557	0.987	0.692	0.978	0.308
	fold change	0.309	0.999	0.968	0.966	0.032

0.5	GLRT	0.945	0.998	0.966	0.997	0.034
	LNN	0.635	0.986	0.873	0.972	0.127
	t-test	0.516	0.968	0.435	0.977	0.565
	fold-change	0.252	1	1	0.966	0

0.9	GLRT	0.980	0.999	0.990	0.999	0.010
	LNN	0.568	0.988	0.877	0.987	0.123
	t-test	0.406	0.972	0.436	0.969	0.564
	fold change	0.139	1	1	0.956	0

### Results from microarray experiments

Any artificial scenario inevitably is biased regarding the underlying model and only reflects certain aspects of biological reality. Therefore, the proposed method is tested in on two real datasets to verify its performance in real world applications. The first dataset contains the gene expression profiles of adenocarcinoma and normal tissues [32]. The data was gathered on the following website . In the microarray experiment, *n *= 18 pairs of colon adenocarcinoma and normal colon samples were studied and *N *= 7457 cDNAs and ESTs are represented on the oligonucleotide array. We apply our method to the microarray data to identify the differentially expressed genes in colon adenocarcinoma. The parameters **α **and Θ are estimated, and the estimate of correlation coefficient is $\hat{\rho }$ = 0.80. The GLRT *κ*_*g*_s of Equation (8) are calculated for the inference controlling FDR *α *= 0.001 and 374 DE genes are identified using our multivariate Bayesian formulation. However, in [32] 47 down-regulated and 19 up-regulated genes in adenocarcinoma are listed whose ratios are more than 4-fold and *p*-values associated was also marginally greater than 0.001. Comparatively, we have discovered that all 47+19 = 66 genes in [32] are detected with high confidence using our method. Furthermore, our gene list also contains many gene products that are related to 66 genes in [32], such as Ckshs2, MGSA, matrilysin, and diverse products related to proliferation and metabolic rate. Some genes related to guanylin and colon mucosa antigen are also identified as significant genes with our model. Therefore, our results include not only the genes that are already known to be expressed abnormally in colon cancer, but also other genes confirmed by biological experiments [32].

The proposed method is also illustrated with another example of microarray data analysis where the objective is to identify differentially expressed genes in mouse liver after treatment with a toxic metal (Cadmium). In our microarray experiment, *n *= 6 hybridizations are repeated for *N *= 1824 genes and we obtained 6 pairs of red and green intensity for each gene, $z_{gi}=(z_{gi}^{red},z_{gi}^{green})'$ (*g *= 1,⋯,1824; *i *= 1,⋯,6). Data normalization is essential and we still denote the normalized data with **z**_*gi *_[33]. As shown in Figures 1 and 2, the error structure of our microarray data depends on the means and correlations between the intensities measured from different dyes. Hence we apply the multivariate Bayesian framework to our microarray data. The parameters **α **and Θ are estimated, in which the estimate of correlation coefficient is $\hat{\rho }$ = 0.92. Then the GLRT *κ*_*g*_s of Equation (8) are calculated for inference and the BH-method is performed to adjust the p-values controlling the FDR *α *= 0.01 in multiple testing. The critical value is calculated to be 10.85, which means the genes in the following set are inferred as DE genes

*J*(*α*) = {*g *: *λ*_*g *_≥ *κ *= 10.85},

Using this criterion, 183 genes are identified. The two-sample t-test detects 44 specific genes controlling the FDR = 0.01 while the fold-change only detects 6 genes. In fact, the above mentioned 6 genes from the fold-change and 44 from the t-test are all included in *J*(*α*). Furthermore, the fold-change does not provide the estimation of the FDR. We have applied another commonly used approach, called the significance analysis of microarray (SAM) [11]. When we adjust the parameters especially Δ to detect 183 genes in which 82% belongs to *J*(*α*), it gives a higher FDR about 2.81% than ours. Comparatively, our method provides a more powerful tool for identification of DE genes while keeping a lower FDR.

Although the DNA microarray technology is very effective for understanding alterations in genome-wide patterns of gene expression, there may be situations in which we need more evidence to determine which genes are truly differentially expressed from the statistical results and further biological analysis may be required to verify the candidate genes. In our study, we also perform another biological test, the reverse-transcription polymerase chain reaction (RT-PCR) to confirm the DE genes. We have found that the relative expression of Ctsc (cathepsin C), Dnase2 (deoxyribonuclease II), Mt-1 (Metallothionein-I) and A2m (alpha-2-macroglobulin) after the normalization are up-regulated in triplicate analysis. Based on gene ontology (GO) analysis [34], they are highly related to the transcriptional regulatory of prostease inhibitor activity (GO: 0030414) and detoxification of copper ion (GO:0010273). This implies that there is a good correlation between the microarray experiment, RT-PCR, as well as the Bayesian method we have proposed.

## Discussion

The DNA microarray technology has important applications in gene expression data analysis. However, the potential sources of random and systematic measurement errors are a critical issue in statistical analysis. It is impossible to propose a statistical model that reflects all sources of errors. Therefore, a good model should capture the most essential features of the data. Currently, the Bayesian methods provide a practical and effective tool for microarray analysis. We have explored the multivariate Bayesian framework to identify DE genes in replicated microarray experiments. More inherent characteristics of expression data are accommodated in the proposed model that is flexible and adaptable to the measurements of each spot. DE genes can be inferred by the GLRT adjusted by BH-method controlling the FDR. In comparison with other methods, the operational characteristics of our method are better than the intuitive fold change, the t-test and the LNN model. Furthermore, our method produces lower FDR and higher efficiency of identification.

Moreover, our model can be extended to the microarray experiments under multiple conditions beyond control and treatment. For example, one may be interested in gene expression of *k *different dosages of some medicine with replicated microarray experiments. That is to say, *k *measurements are observed from one gene spot. If there are *N *genes on one chip and *n *hybridizations are repeated, the measurements from one gene spot are written as $z_{gi}=(z_{gi}^{1},z_{gi}^{2},\cdots ,z_{gi}^{k})'$ (*g *= 1,⋯,*N*; *i *= 1,⋯,*n*). The corresponding Bayesian model, similar to Equations (1) and (2) can also be applied to model the expression data, and only the dimensionality of the feature vectors is k instead of 2. Besides the global parameter **α**, the gene-specific parameters are denoted as

$$\mu_{g}^{0}=(\mu_{g1}^{0},\mu_{g2}^{0},\cdots ,\mu_{gk}^{0})'$$

and

$$\Lambda_{0}=\left(\begin{array}{cccc} \Lambda_{11}^{0} & \Lambda_{12}^{0} & \cdots & \Lambda_{1k}^{0}\\ \Lambda_{21}^{0} & \Lambda_{22}^{0} & \cdots & \Lambda_{2k}^{0}\\ \vdots & \vdots & \ddots & \vdots \\ \Lambda_{k1}^{0} & \Lambda_{k2}^{0} & \cdots & \Lambda_{kk}^{0} \end{array}\right)$$

As to the inference, the number of hypotheses would increase significantly with the number of conditions. For example, there are 5 hypotheses to infer under 3 conditions, equivalent expression, altered expression in one condition and distinct expression in each condition. Thus, only some patterns of interest should be tested with the GLRT calculated on the specific constraints.

With the widespread applications to microarray data analysis, more sophisticated Bayesian methods are needed to solve more statistical problems, such as normal assumption, gene independence and parameter estimation. Normality and independence are regarded as the devices deducing the probability distribution function, but we believe more improvement can be made, especially in terms of dependence.

## Conclusions

We have presented a multivariate Bayesian model for differential gene expression data analysis. In addition to the gene-specific variance, this model takes into account the covariance between the pair of measurements to relax the constant assumption of correlation coefficient in the common used hierarchical models. Our model provides a more realistic and flexible estimate for the variance of gene expression data under limited replicates. Based on the multivariate hierarchical model, the multiple GLRT takes into account the power of gene-specific variance, latent gene variance and covariance. In our examples above, the results obtained from our model show better operating characteristics, especially when the correlation coefficient of gene expression within one spot is significant. This indicates that the power of identification of differentially expressed genes can be improved if the Bayesian model is developed in accordance with the statistical properties of microarray data.

## Methods

### Toxic microarray experiment

Cadmium (Cd) is a ubiquitous environmental toxic pollutant with a well established toxicity. Chronic exposure or even low concentration of Cd has been shown to result in a variety of pathological disorders such as cancers, anemia, osteoporosis, renal and hepatic dysfunction. A microarray experiment was designed in the biomedical laboratory of the department of biology, Hong Kong Baptist University. They explore the genes that are differentially expressed with the toxic treatment, using duel colors (Cy3 and Cy5) DNA microarray to compare the treatment group with CdCl_2 _and control group with NaCl. In our microarray experiment, eight male adult mice, ICR strain, were randomly separated into four groups and denoted as C1T1 and C2T2. In each group one serves as the control which the other one is for treatment. The mice in control and treatment group were given a single intraperitoneal injection of 0.3 ml 0.9% NaCl or the same volume of 2 mg/kg CdCl2 respectively. After 48 hours, mice were sacrificed and the livers were collected. Total RNA were extracted from each liver using the Trizol reagent. The total RNA samples were reverse transcribed to cDNA in the presence of fluorescent (Cy3) or (Cy5) dye. Usually, the treatment group is labelled with Cy3 while the control group is labelled with Cy5. Probes were then hybridized onto the UCLA M07 microarray arrays overnight at 65°*C*. After two subsequent washings in 2 × SSC, 0.1% SDS and 0.2 × SSC buffer, all the hybridized chips were scanned using ScanArray 5000 confocal laser scanner (Packard BioChip BioScience Technology) and images were further analyzed by the QuantArray Quantitative Microarray Analysis Software. C1T1 and C2T2 groups were tested in three individual hybridization experiments and thus 6 hybridized chips are replicated measured. After image analysis, there is one pair of red and green fluorescence intensities (after background correction) observed from each spot, and 6 replicated pairs for each gene. We applied the logarithm transformation to the measurements as commonly used in microarray analysis. Before statistical analysis, the microarray data have to be normalized and filtered to removing some variation of expression levels in fluorescence intensities [32]. After data processing, we denote *n *replicated pairs of observation component of *g*th gene as **Z**_*g *_= (**z**_*g*1_,⋯,**z**_*gn*_)', in which the green and red log-intensities of *g*th gene of *i*th replication is **z**_*gi *_= (*x*_*gi*_, *y*_*gi*_)' (*g *= 1,⋯,1824; *i *= 1,⋯,6).

### Lognormal-Normal (LNN) model

The parametric Bayesian model is characterized by mixing measurements over a latent gene-specific component *π*(*μ*_*g*_, $\sigma_{g}^{2}$). In the LNN model [26], the measurements *x*_*gi *_and *y*_*gi *_(*g *= 1,⋯,*N*; *i *= 1,⋯,*n*) can be expressed in terms of the observation components following a normal distribution

$$\begin{array}{cc} x_{gi}|\mu_{g},\sigma_{g}^{2}\sim N(\mu_{g},\sigma_{g}^{2}) & y_{gi}|\mu_{g},\sigma_{g}^{2}\sim N(\mu_{g},\sigma_{g}^{2}) \end{array}$$

and the hierarchical gene-specific components

$$\begin{array}{cc} \mu_{g}|\sigma_{g}^{2}\sim N(\mu_{0},\sigma_{g}^{2}/\lambda_{0}) & \sigma_{g}^{2}\sim IG(\nu_{0},\sigma_{0}^{2}) \end{array}$$

where IG denotes the inverse Gamma (IG) distribution and *μ*_0_, *λ*_0_, *ν*_0_, $\sigma_{0}^{2}$ are the hyperparameters. Notice that the dependence between *μ*_*g *_and $\sigma_{g}^{2}$ is implied with the conjugate prior *π*(*μ*_*g*_, $\sigma_{g}^{2}$) whose posterior probability has the same functional form. All measurements *x*_*gi *_and *y*_*gi *_in this framework are assumed to arise independently and identically from the same distributional class.

### Multiple testing

The error rate in hypothesis testing can be summarized in Table 2. In the microarray context, the specific *N *hypotheses is known to be the number of genes on one array; *R*_0 _and *R*_1 _(*R*_0_+*R*_1_= *N*) are observable random variables; *N*_0 _and *N*_1 _(*N*_0 _+ *N*_1 _= *N*) are unknown parameters; and others are unobservable random variables. In general, one would like to minimize type I errors, false positives (FP), and type II errors, false negatives (FN) [9,27].

**Table 2:** Number of errors in *N *multiple test

	# not rejected (negative)	# rejected (positive)	Total
# True H_0 _(EE)	*TN*	*FP*	*N*_0_
# Non-true H_0 _(DE)	*FN*	*TP*	*N*_1_
	*R*_0_	*R*_1_	*N*

# denotes the number of elements in the set.

In microarray analysis, the FDR is defined as the expectation of the ratio of rejected null hypotheses which are erroneously rejected, that is, the average of the ratio of the number of false positives to the number of genes identified as DE. Because of typical large *N *and small *n *in microarray data, the type I errors increase when many hypothesis are tested and each test has a specified type I error probability.

Obviously, it is intuitive to test in the univariate setting to minimize type II errors rates under the prespecified type I error rate. As to the case under multiple testing, we have different procedures. Some definitions about type I error rate are described, such as FDR, FWER or PCER in [11]. Benjamini and Hochberg's *p*-value adjustment provided a more powerful procedure to control FDR [9,11,27,30,31]. Based on the approximate *χ*^2 ^distribution of *κ*_*g*_, we can apply the method for the significant testing to identify the DE genes. The algorithm of theBH-method is described as:

Step 1: Order the *p*-value corresponding to testing *N *hypotheses of *H*_*g*0 _: *p*_(1) _≤ *p*_(2) _≤ ⋯ *p*_(*N*)_.

Step 2: Define the Bonferroni type multiple-testing $p_{\left(g\right)}\leq \frac{g}{N}\alpha$, where *α *is the value of the controlled FDR. Let *m *be the largest *g *to satisfy the inequations.

Step 3: Reject all *H*_(*g*)0 _(*g *= 1,2,⋯,*m*), that is to say, genes indexed by (1), (2),...,(*m*) might be identified as the DE genes.

Besides the FDR in the multiple testing, there are other statistics to assess the significance [9,27]. In microarray data analysis, sensitivity is defined as the fraction of the true DE genes correctly identified as DE, i.e. *TP/N*_1_; specificity is defined as the fraction true EE genes correctly identified as EE, i.e. *TN/N*_0_; PPV of H_gk _is the fraction of the DE genes that give a positive result, i.e. *TP/R*_1_; and NPV is *TN/R*_0_.

## Competing interests

The authors declare that they have no competing interests.

## Authors' contributions

Hongya Zhao designed and implemented the algorithms, analyzed the data, and drafted the manuscript. Kwok Leung Chan, Lee-Ming Cheng and Hong Yan conceived the project, gave suggestion to improve the algorithm, and assisted in drafting and editing the manuscript. All authors have read and approved the final manuscript.
